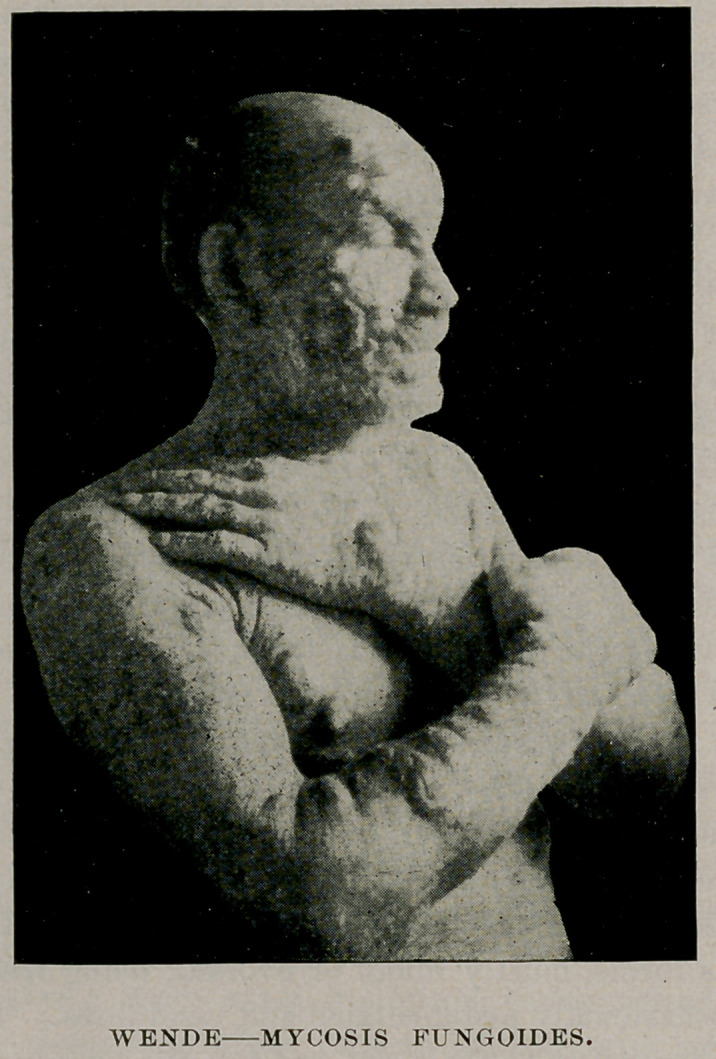# Mycosis Fungoides; with a Report of Case1Read at the pathological section Buffalo Academy of Medicine, May 18, 1897.

**Published:** 1897-06

**Authors:** Grover William Wende

**Affiliations:** Instructor of dermatology, University of Buffalo, 471 Delaware Avenue


### MYCOSIS FUNGOIDES; WITH A REPORT OF CASE.[1]

BY GROVER WILLIAM WENDE, M. D.,
Instructor of dermatology, University of Buffalo.

IN THE year 1814, Alibert, under the term of mycosis fungoides, recorded a condition which he depicted as certain mulberry masses, prone to ulceration, which he regarded as syphilitic. However, in a later contribution, published in 1833, he defined it as a distinct pathological process. Since then the malady has been recognised by numerous dermatologists under as many different names, but so loosely and carelessly employed that it was difficult to understand the precise meaning which these terms were intended to convey. The Germans suggested the name of granuloma fungoides; the French preferred to call it lymphadëmie cutanée; the English, not so conceited in their nomenclature, described the morbid state as an eczema hypertrophicum, or tuberosum, or as a fibrome fungoides; while the Americans, not to be excelled in confusion and uncertainty, have characterised the disease by numerous pseudonyms. Duhring, to secure clearness and accuracy, called it inflammatory fungoid neoplasm; Van Harlingen, hoping to be rightly understood, styled it ulcerated scrofuloderma; and Hyde, thinking to give a more exact definition, characterised it as myeloma cutis. Language often fails to express with precision the ideas which one conceives of things and this is especially true in medicine. But in order to avoid perplexity, an agreement of views is absolutely necessary. Therefore it is desirable in cases like the present that a single name should be adopted, the others following it as synonyms.

When the square of the hypotenuse is geometrically demonstrated no one has a right to his own notion about it. Until, then, a better name is found, let that of mycosis fungoides stand in honor of Alibert, who first called our attention to this distressing, but interesting, disease.

---

[1] Read at the pathological section Buffalo Academy of Medicine, May 18, 1897.

The disease comprises three distinct stages, although all of them may not necessarily be present in each individual case. Some latitude must be allowed to the characteristics of these various stages.

1. The eczematoid stage is accompanied by a course which may be either brief or protracted, in some instances lasting only a few weeks, in others lingering for several years. Its cutaneous manifestations are numerous, varying greatly in size, form and character and representing the different types, as simple erythema, eczema, urticaire and psoriasis, which may be located anywhere on the body and which are usually attended by an intense pruritus.

2. The lichenoid stage is formed of eczematoid or psoriasiform plaques, which involve small, dense, lentil-sized papules, lasting for a varying period and then ostensibly melting away, only to be followed by others. They frequently affect the healthy skin, in which case they are apparently sub-epidermic and are usually of uniform color. They, however, present various hues, from bright red to reddish blue or purple. They also have a shiny appearance. In shape, as the disease progresses, they become elevated and nodular. The itching accompanying the disease is severe, and from repeated rubbing the hair becomes thinned. The duration of this stage varies from a few months to two years.

3. The fungoid stage succeeds when the pathological process concentrates into tubercles, nodules and tumors, as a sequence of the lichenoid stage. However, in extraordinary instances this particular stage may assume the initiative and mark the incipiency of the outbreak. In the development of the growths just named they take various forms, mostly round or ovoidal, being frequently flattened and occasionally pedunculated and pendulous. They vary in consistence according to their size and age, the younger and smaller ones being usually quite firm and the older and larger ones quite soft and doughy. Their integument may at first be natural in color, but, later, it becomes more or less livid. The character of the skin is so modified, in some instances, as to be barely recognisable. The tumors disappear by absorption or ulcerate superficially. Fresh nodes frequently form on the site occupied by the tumors which have disappeared. Thus the difficulty steadily progresses until life terminates from sheer exhaustion or some intercurrent disease.

CASE.—Frederick B., the subject of the case which I report this evening, was at the time of his death 55 years old. He was born in Germany and lived there until he attained the age of 6, when he came to this country, settled in Tonawanda, N. Y., and there remained until his death. For the most of his life he kept a saloon. His family and personal history, with the exception of an occasional attack of rheumatism, were good. There is no record of syphilis. He was five feet nine inches in height, of excellent proportions and weighed about 150 pounds. He had four healthy children. His mother died at the age of 90. His

WENDE—MYCOSIS FUNGOIDES.

father was accidentally suffocated by gas at the age of 75. His two sisters are still living and are in excellent health. Five years before his death and just prior to the appearance of the disease which finally proved fatal, he visited Mount Clemens to seek relief from an attack of rheumatism. Instead, however, of obtaining help by the use of the waters, his sufferings were thereby increased. The skin over limited areas of the back and shoulders became irritable and itched and his gait was unsteady. Immediately upon his return home he consulted Dr. Murray, his family physician, who, judging from his irregular and peculiar locomotion, regarded the ailment as locomotor ataxia. The difficulty lasted four months and was treated with electricity. The initial cutaneous manifestations were confined to the scapular region and were pruriginous in character, as evinced by scratch-marks. This locality, however, soon showed well-developed, slightly scaly patches, which for two and a half years were evanescent. Finally, the remissions grew less frequent and their duration shorter, while the patches became more diffused, so that in a short time the surface of the entire body had gradually undergone a change and was more or less affected by the same scaly patches. It was a notable circumstance that the lichenoid stage was wanting; at least no lesions which harmonised with it had been developed, the mycotic stage being ushered in by an almost simultaneous production of numerous nodes and tumors which continuously appeared upon and disappeared from the various portions of the body. Sometimes great numbers were manifested in rapid succession. The first "lump" (as the patient expressed it) to show itself was situated on the outer side of the left leg, which was immediately followed by a similar one on the right leg in the corresponding locality. Others rapidly succeeded, so that, in a short time, they were visible in every stage of development, with wide diversity of shape and size. The face, body, arms, legs, hands, feet, fingers and toes were studded with them. Upon the right cheek they coalesced and produced an ulceration of some magnitude. The back and chest were the only regions partially involved. The course and development of each individual tumor, or node, varied greatly. Some were acute, others were chronic; and as they increased in size they *became* softer and took on a mushroom-like appearance. A certain number of them were much darker at the base than at the apex, and the entire growth was overflowed with a profuse gelatinous exudation. Some grew to the dimensions of a moderate-sized orange, while others remained stunted, not being larger than a pea. Upon the trunk they were flattened and strikingly elevated, while upon the face, arms and legs they exhibited an oval contour. The discharge was sero-purulent in character and possessed a pungent and offensive odor. Several of the neoplasms which were conspicuous early in the course of the disease had faded completely, leaving a pigmented aspect, while others had only partially disappeared. Accessory to all these new pathological formations, the surface of the body revealed an endless number of plaques, some that glistened from a serous exudation; some that were reddened from a hyperemia; and others that became squamous from a tendency toward improvement, taking on either the cast of eczema, erythema or psoriasis. At the time of death there were over 500 of these different-sized tumors situated upon the various portions of his body; and ten days earlier those about the orbit became necrotic in their interior. Coincident with this unusual feature may be recorded the additional interesting fact of total blindness. The face was also much swollen. During the whole course of the patient's illness, with the exception of the two weeks preceding his death, the irritation of the skin was distressing. The lymphatic glands were greatly enlarged, notably the inguinal, axillary and post-cervical. Immediately after blindness came upon him his condition began to give evidence of near dissolution. He grew suddenly weaker, there was rapid emaciation, and death soon terminated his sufferings.

As the patient lived out of town my opportunity for observation and investigation was limited. An autopsy was positively refused. However, I procured, accidentally or otherwise, a small section of one of the tumors which histologically demonstrated a reticulated tissue, filled with oval cells densely packed, corresponding in size with the white blood corpuscles. I discovered but few capillaries. On the whole, the slide resembled the microscopic appearance of a lympho-sarcoma.

Regarding the treatment, such remedies as arsenic, iron, ergot, potassium iodid, and the like, were carefully administered ; locally, ichthyol, salicylic acid, resorcin, carbolic acid, terebine and the like, were employed. The means, however, proved inadequate and ineffectual.

471 DELAWARE AVENUE.

## Figures and Tables

**Figure f1:**